# Comparison of Sequential Organ Failure Assessment (SOFA), Quick Sequential Organ Failure Assessment (qSOFA), Obstetrically Modified SOFA, and Obstetrically Modified qSOFA Scores for Predicting Maternal Outcomes in High-Risk Obstetric Patients

**DOI:** 10.7759/cureus.115985

**Published:** 2026-09-09

**Authors:** Monika Chaudhary, Divya Yadav, Nidhi Gupta, Arpita Saxena, Asha Nigam, Neelam Singh

**Affiliations:** 1 Obstetrics and Gynaecology, Sarojini Naidu Medical College, Agra, IND; 2 Obstetrics and Gynecology, Sarojini Naidu Medical College, Agra, IND; 3 Anaesthesiology, Sarojini Naidu Medical College, Agra, IND

**Keywords:** high-risk pregnancy, intensive care, maternal mortality, maternal outcomes, obstetrically modified sofa, obstetric critical care, organ dysfunction, prognostic scoring systems, qsofa, sofa score

## Abstract

Introduction

Maternal morbidity and mortality remain significant challenges in obstetric critical care. Early identification of organ dysfunction is essential for timely intervention and improved outcomes. This study compared the prognostic performance of the Sequential Organ Failure Assessment (SOFA), quick Sequential Organ Failure Assessment (qSOFA), obstetrically modified SOFA (omSOFA), and obstetrically modified qSOFA (omqSOFA) scores in predicting maternal outcomes among high-risk obstetric patients.

Methods

A prospective observational study was conducted among 200 high-risk obstetric patients admitted to a tertiary care center. Twelve patients who left against medical advice were excluded from outcome-based analyses, leaving 188 patients for final evaluation. SOFA, qSOFA, omSOFA, and omqSOFA scores were calculated at admission. Receiver operating characteristic (ROC) curve analysis was performed to determine the predictive performance of each scoring system for maternal mortality. Area under the curve (AUC), sensitivity, specificity, positive predictive value (PPV), negative predictive value (NPV), and optimal cutoff values were calculated.

Results

Among the 188 patients included in the final analysis, 15 maternal deaths occurred. SOFA demonstrated the highest discriminatory ability for predicting maternal mortality, with an AUC of 0.97 (95% CI: 0.94-0.99) at a cutoff value of ≥10, followed by omSOFA with an AUC of 0.93 (95% CI: 0.89-0.97) at a cutoff value of ≥9. qSOFA and omqSOFA showed AUCs of 0.76 (95% CI: 0.68-0.84) and 0.79 (95% CI: 0.71-0.87), respectively. qSOFA and omqSOFA demonstrated 100% sensitivity and negative predictive value at a cutoff score of ≥2, while SOFA and omSOFA provided superior specificity and overall discriminatory performance.

Conclusions

Among high-risk obstetric patients, SOFA demonstrated the highest discriminatory ability for predicting maternal mortality, followed by omSOFA. While qSOFA and omqSOFA showed lower specificity, they demonstrated excellent sensitivity and negative predictive value, making them useful bedside screening tools for early risk identification. Incorporation of these scoring systems into obstetric critical care practice may facilitate timely risk stratification and early intervention. Further multicentre studies are required to validate these findings in larger and more diverse obstetric populations.

## Introduction

Maternal sepsis remains an important cause of maternal morbidity and mortality worldwide and continues to pose a significant challenge in obstetric practice. Early recognition of clinical deterioration and organ dysfunction is essential for timely management and improvement of maternal outcomes [1,2]. The Sequential Organ Failure Assessment (SOFA) score was developed to quantify the degree of organ dysfunction in critically ill patients and has subsequently been widely used for prognostic assessment in intensive care settings [3,4]. The Third International Consensus Definitions for Sepsis and Septic Shock (Sepsis-3) incorporated SOFA-based organ dysfunction into the contemporary definition of sepsis and introduced the quick Sequential Organ Failure Assessment (qSOFA) as a simplified bedside assessment for identifying patients at increased risk of poor outcomes [5].

Application of these scoring systems to obstetric patients presents particular challenges because the physiological adaptations of pregnancy may influence several clinical and laboratory parameters used in conventional critical care assessment [6,7]. To address these differences, pregnancy-adapted versions of the scores-obstetrically modified SOFA (omSOFA) and obstetrically modified qSOFA (omqSOFA)-have been proposed. These modifications incorporate pregnancy-appropriate thresholds for the assessment of organ dysfunction and clinical deterioration [7,8]. However, available evidence on the performance of conventional and obstetrically modified scoring systems remains limited, and further validation in pregnant and postpartum populations is required.

Maternal sepsis guidelines and obstetric early-warning approaches emphasize prompt recognition of deterioration, structured assessment, and timely treatment [7,9,10]. Nevertheless, the relative prognostic performance of conventional SOFA and qSOFA compared with their obstetrically modified counterparts in critically ill obstetric populations remains incompletely established. The present study aimed to compare the prognostic performance of SOFA, qSOFA, obstetrically modified SOFA (omSOFA), and obstetrically modified qSOFA (omqSOFA) in predicting maternal mortality among high-risk pregnant and postpartum women requiring high dependency unit (HDU) or intensive care unit (ICU) care.

## Materials and methods

Study design and setting

A prospective observational study was conducted in the Department of Obstetrics and Gynaecology at Sarojini Naidu Medical College and Associated Hospitals, Agra, India. A preliminary pilot/feasibility phase was undertaken before formal enrollment to assess the feasibility of data collection and application of the proposed scoring systems; data from this phase were not included in the final study cohort or analysis. Formal prospective enrollment commenced after approval (Approval No. SNMC/IEC/DHR/2024/82) from the Institutional Ethics Committee on October 21, 2024, and continued until October 2025. Written informed consent was obtained from all participants.

Study population

A total of 200 high-risk obstetric patients were prospectively enrolled after ethical approval. The study population comprised pregnant women and women within 42 days postpartum requiring HDU or ICU care because of severe obstetric or medical complications. The major indications for HDU/ICU admission included hypertensive disorders of pregnancy, obstetric hemorrhage, sepsis/infection, cardiac disease, and other severe obstetric and medical complications. Their distribution and frequencies are reported in the Results section.

Inclusion and exclusion criteria

Pregnant women and women within 42 days postpartum admitted to the HDU or ICU with severe obstetric or medical complications requiring intensive monitoring were included in the study. Patients with incomplete clinical or laboratory data required for calculation of the study scores, those who left against medical advice (LAMA), and those with missing outcome information were excluded from the final analysis. Of the 200 enrolled participants, 12 left against medical advice before outcome assessment; therefore, 188 patients were included in the final outcome and scoring-system analyses.

Scoring systems

Four scoring systems were evaluated at admission: Sequential Organ Failure Assessment (SOFA), quick Sequential Organ Failure Assessment (qSOFA), obstetrically modified SOFA (omSOFA), and obstetrically modified qSOFA (omqSOFA). The SOFA score assessed dysfunction across six organ systems: respiratory function using the partial pressure of arterial oxygen (PaO₂)/fraction of inspired oxygen (FiO₂) ratio, coagulation using platelet count, hepatic function using serum bilirubin, cardiovascular function using mean arterial pressure and vasopressor requirement, neurological function using the Glasgow Coma Scale (GCS), and renal function using serum creatinine or urine output. Each organ system was scored from 0 to 4, for a total score ranging from 0 to 24, according to the established SOFA criteria described by Vincent et al. (1996) [3] and Vincent et al. (1998) [4].

The qSOFA score consisted of three bedside clinical parameters: respiratory rate ≥22 breaths/min, systolic blood pressure ≤100 mmHg, and altered mental status (GCS <15). One point was assigned for each criterion, resulting in a total score ranging from 0 to 3. qSOFA was calculated according to the previously published criteria of Seymour et al. [11]. The omSOFA score retained the six-organ framework of the conventional SOFA score but incorporated pregnancy-adapted thresholds to account for physiological changes occurring during pregnancy and the postpartum period. The omqSOFA score similarly used pregnancy-adapted clinical thresholds for rapid bedside assessment. The pregnancy-specific modifications were applied according to the obstetric scoring criteria described in the referenced literature [8]. 

Outcome measures

The primary outcome was maternal mortality. Secondary outcomes included requirement for ICU-level care, prolonged hospitalization, and severe maternal morbidity. Prolonged hospitalization was defined as hospitalization beyond seven days. Requirement for ICU-level care was defined as transfer or escalation to the ICU for invasive monitoring, organ support, or intensive therapeutic interventions, including mechanical ventilation, vasopressor support, or renal replacement therapy. Severe maternal morbidity included major complications such as septic shock, acute respiratory distress syndrome, acute kidney injury, disseminated intravascular coagulation, organ failure, and requirement for mechanical ventilation.

Statistical analysis

Statistical analysis was performed using IBM SPSS Statistics version 26.0. Continuous variables were expressed as mean ± standard deviation or median with interquartile range, as appropriate, and categorical variables as frequencies and percentages. Continuous variables were compared using Student's t-test or Mann-Whitney U test, and categorical variables using the chi-square test or Fisher's exact test, as appropriate.

Receiver operating characteristic (ROC) curve analysis was performed to assess the ability of SOFA, qSOFA, omSOFA, and omqSOFA to predict maternal mortality. The area under the curve (AUC), sensitivity, specificity, positive predictive value (PPV), and negative predictive value (NPV) were calculated. Optimal cutoff values were determined by maximizing the Youden index (J = sensitivity + specificity − 1). Ninety-five percent confidence intervals were calculated for diagnostic performance estimates. Missing data were handled using complete-case analysis. A p-value <0.05 was considered statistically significant. Formal pairwise statistical comparison of the ROC curves was not performed; therefore, differences between AUCs were interpreted descriptively.

Sample size

A sample size of 200 participants was calculated using the formula for estimating a proportion with finite population correction, assuming an expected prevalence of adverse outcomes of approximately 30%-40%, a 95% confidence level, and an allowable error of 7%. The calculation was based on overall adverse outcomes and was not specifically powered for maternal mortality.

## Results

Baseline characteristics

A total of 200 high-risk obstetric patients were enrolled in the study. Twelve patients who left against medical advice before outcome assessment were excluded from outcome-based analyses, leaving 188 patients for the final evaluation. The baseline demographic and clinical characteristics of the study population are presented in Table 1.

**Table 1:** Baseline demographic and clinical characteristics of the study population (N = 200)

Characteristic	Category	N (%)
Age (years)	<20	9 (4.5)
	20–25	44 (22.0)
	26–30	88 (44.0)
	31–35	44 (22.0)
	>35	15 (7.5)
Parity	Primipara	50 (25.0)
	Para 2	94 (47.0)
	Para ≥3	56 (28.0)
Booking status	Booked	72 (36.0)
	Unbooked	128 (64.0)
Obstetric status	Antepartum	76 (38.0)
	Postpartum	88 (44.0)
	Post-abortal	36 (18.0)
Indication for admission	Sepsis	96 (48.0)
	Hypertensive disorders	44 (22.0)
	Obstetric hemorrhage	22 (11.0)
	Respiratory disease	22 (11.0)
	Coagulation disorders	8 (4.0)
	Cardiac disease	6 (3.0)
	Other medical disorders	2 (1.0)

Indications for HDU/ICU admission

The study population represented a heterogeneous spectrum of obstetric and medical conditions requiring HDU/ICU care. Severe preeclampsia/eclampsia was the most common indication for admission (16%). Other major indications included obstetric hemorrhage, sepsis/infection, cardiac disease, and other severe obstetric and medical complications.

Secondary outcomes

Among the study population, 78 patients (39%) required ICU-level care, while 55% had prolonged hospitalization beyond seven days. Major maternal morbidities included septic shock (9.5%), acute respiratory distress syndrome (9.5%), mechanical ventilation (8.5%), acute kidney injury (8%), and disseminated intravascular coagulation (5%). These findings demonstrate the spectrum of severe maternal morbidity observed in the study cohort. 

Receiver operating characteristic (ROC) curve analysis was performed to evaluate the ability of the four scoring systems to predict maternal mortality. SOFA had the numerically highest AUC of 0.97 (95% CI: 0.94-0.99), followed by omSOFA with an AUC of 0.93 (95% CI: 0.89-0.97). qSOFA and omqSOFA showed AUC values of 0.76 (95% CI: 0.68-0.84) and 0.79 (95% CI: 0.71-0.87), respectively. Detailed ROC analysis, including optimal cutoff values, sensitivity, specificity, positive predictive value, negative predictive value, and Youden index, is shown in Table 2. The ROC curves for all four scoring systems are illustrated in Figure 1.

**Table 2:** Receiver operating characteristic (ROC) analysis of SOFA, qSOFA, omSOFA, and omqSOFA scores for predicting maternal mortality AUC, area under the curve; CI, confidence interval; PPV, positive predictive value; NPV, negative predictive value; SOFA, Sequential Organ Failure Assessment; qSOFA, Quick Sequential Organ Failure Assessment; omSOFA, obstetrically modified Sequential Organ Failure Assessment; omqSOFA, obstetrically modified Quick Sequential Organ Failure Assessment.

Scoring System	AUC (95% CI)	Optimal Cutoff	Sensitivity (%)	Specificity (%)	PPV (%)	NPV (%)	Youden Index
SOFA	0.97 (0.94–0.99)	≥10	93.3	89.7	42.4	99.4	0.830
qSOFA	0.76 (0.68–0.84)	≥2	100.0	54.1	15.8	100.0	0.541
omSOFA	0.93 (0.89–0.97)	≥9	93.3	82.2	29.8	99.3	0.755
omqSOFA	0.79 (0.71–0.87)	≥2	100.0	56.8	16.7	100.0	0.568

**Figure 1:**
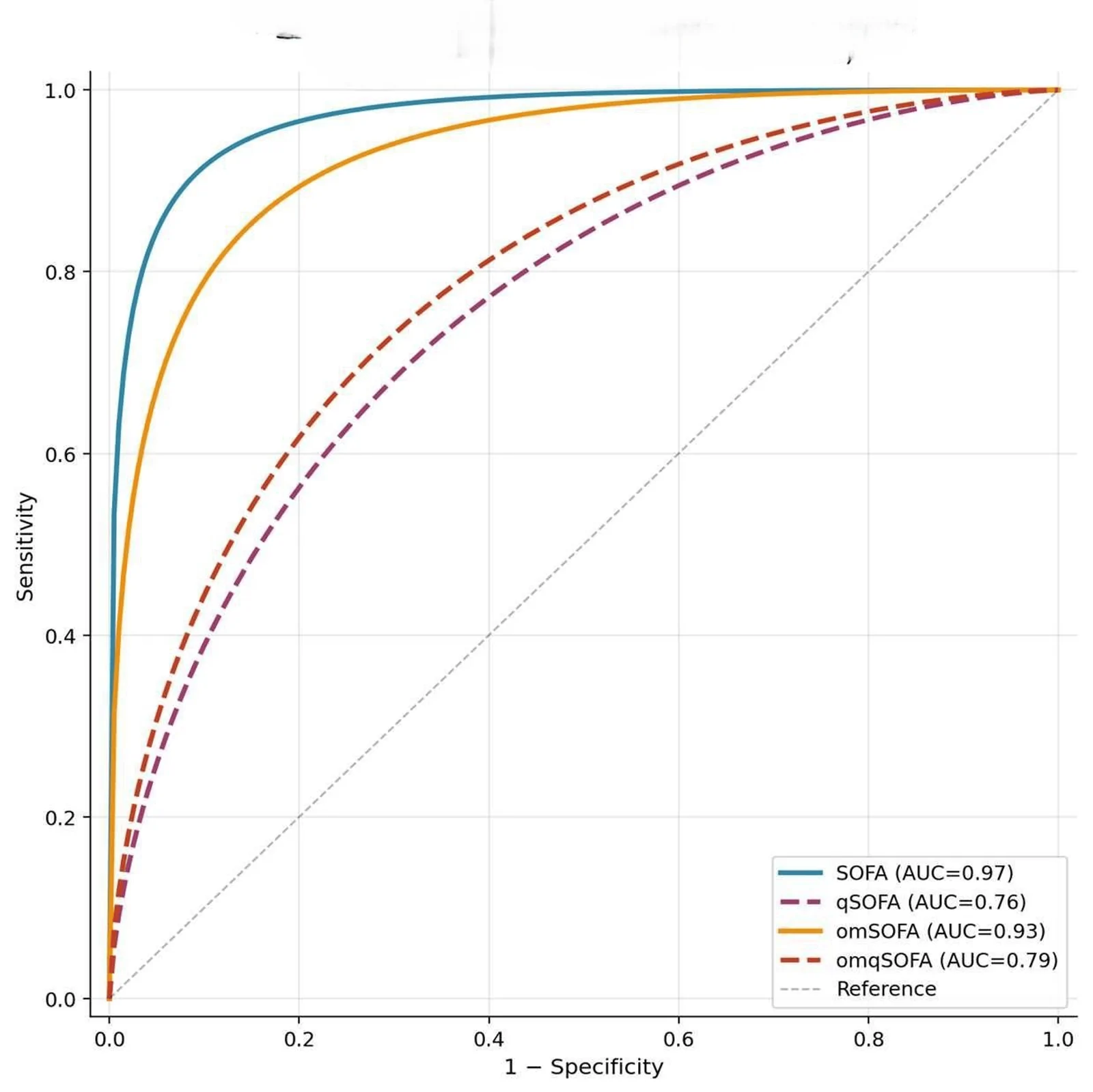
ROC curves of SOFA, qSOFA, obstetrically modified SOFA, and obstetrically modified qSOFA scores for predicting maternal mortality Receiver Operating Characteristic (ROC) curves demonstrating the predictive performance of SOFA, qSOFA, omSOFA, and omqSOFA scores for maternal mortality among high-risk obstetric patients. SOFA demonstrated the highest discriminatory ability (AUC = 0.97), followed by omSOFA (AUC = 0.93), omqSOFA (AUC = 0.79), and qSOFA (AUC = 0.76).

Maternal mortality

Among the 188 patients included in the final analysis, 15 maternal deaths occurred. At the identified optimal cutoffs, SOFA and omSOFA demonstrated sensitivities of 93.3%, with specificities of 89.7% and 82.2%, respectively. qSOFA and omqSOFA achieved 100% sensitivity and negative predictive value at a cutoff score of ≥2, with lower specificities of 54.1% and 56.8%, respectively. Comparative diagnostic performance of the four scoring systems is summarized in Table 3.

**Table 3:** Comparative diagnostic performance of SOFA, qSOFA, omSOFA, and omqSOFA scores for predicting maternal mortality PPV, positive predictive value; NPV, negative predictive value; SOFA, Sequential Organ Failure Assessment; qSOFA, Quick Sequential Organ Failure Assessment; omSOFA, Obstetrically Modified Sequential Organ Failure Assessment; omqSOFA, Obstetrically Modified Quick Sequential Organ Failure Assessment. Optimal cutoff values were determined using the maximum Youden Index obtained from receiver operating characteristic (ROC) curve analysis.

Scoring System	Optimal Cutoff	Sensitivity (%)	Specificity (%)	PPV (%)	NPV (%)
SOFA	≥10	93.3	89.7	42.4	99.4
qSOFA	≥2	100.0	54.1	15.8	100.0
omSOFA	≥9	93.3	82.2	29.8	99.3
omqSOFA	≥2	100.0	56.8	16.7	100.0

## Discussion

The present study evaluated the prognostic performance of SOFA, qSOFA, obstetrically modified SOFA (omSOFA), and obstetrically modified qSOFA (omqSOFA) scores in high-risk obstetric patients. Among the four scoring systems, SOFA had the numerically highest AUC for predicting maternal mortality (0.97), followed by omSOFA (0.93), while qSOFA and omqSOFA had lower AUCs of 0.76 and 0.79, respectively. As formal pairwise comparisons between the ROC curves were not performed, these differences should be interpreted descriptively and do not establish statistical superiority of one scoring system over another.

The high discriminatory performance observed with SOFA is consistent with previous investigations. Vincent et al. originally developed the SOFA score to quantify organ dysfunction and demonstrated its association with adverse clinical outcomes and mortality in critically ill patients [3]. Subsequently, de Grooth et al. reported that SOFA-based endpoints correlate with mortality outcomes and remain valuable prognostic indicators in critical care settings [5]. Similarly, Raith et al. demonstrated greater prognostic accuracy of SOFA compared with qSOFA and SIRS criteria for predicting in-hospital mortality among patients [12]. The present findings suggest that SOFA may also have prognostic value in high-risk obstetric patients; however, further validation in larger obstetric populations is required.

Although qSOFA and omqSOFA demonstrated lower overall discrimination, both achieved 100% sensitivity and negative predictive value at a cutoff score of ≥2 in the present cohort. These findings should be interpreted cautiously because only 15 maternal deaths occurred and the cutoff values were derived and evaluated in the same cohort. The high sensitivity and NPV therefore suggest potential screening value, but are insufficient to establish clinical utility or support their use for referral or escalation-of-care decisions. Previous studies have similarly evaluated qSOFA as a simplified bedside assessment, with variable performance compared with more comprehensive scoring systems [7,9]. The Sepsis-3 consensus proposed qSOFA as a bedside prompt to identify patients with suspected infection who may be at increased risk of poor outcomes [10].

Pregnancy-related physiological changes can influence several clinical and laboratory parameters incorporated into conventional critical care scoring systems. Consequently, obstetrically modified scoring systems have been proposed to account for pregnancy-specific physiological differences. In the present study, omSOFA demonstrated a high AUC of 0.93, numerically approaching that observed with conventional SOFA. Aarvold et al. also evaluated organ dysfunction-based assessment in critically ill obstetric patients and demonstrated the potential value of pregnancy-adapted approaches [8]. Current recommendations for maternal sepsis management similarly emphasize structured clinical assessment and early recognition of organ dysfunction [10]. Nevertheless, the present findings do not establish that obstetrically modified scores are superior to conventional scores, and further comparative validation is required.

A comparison of the present findings with previously published studies is shown in Table 4. Overall, the findings suggest that SOFA and omSOFA have potential prognostic value for mortality risk assessment in high-risk obstetric patients. The simplified qSOFA and omqSOFA scores may also have potential as bedside screening tools; however, their clinical utility cannot be established from the present study and requires validation in larger independent cohorts.

**Table 4:** Comparison of the present study with previously published studies SOFA, Sequential Organ Failure Assessment; qSOFA, Quick Sequential Organ Failure Assessment; omSOFA, Obstetrically Modified Sequential Organ Failure Assessment; omqSOFA, Obstetrically Modified Quick Sequential Organ Failure Assessment; SIRS, Systemic Inflammatory Response Syndrome; MODS, Multiple Organ Dysfunction Score; ICU, intensive care unit; LMICs, low- and middle-income countries; AUC, area under the curve; NPV, negative predictive value.

Study	Population	Scoring systems evaluated	Key findings	Comparison with present study
Vincent et al. (1996) [3], Vincent et al. (1998) [4]	Critically ill ICU patients	SOFA	SOFA was associated with organ dysfunction and mortality	Supports the prognostic value of SOFA observed in the present study
de Grooth et al. [5]	Systematic review of critically ill patients	SOFA	SOFA-based endpoints demonstrated an association with mortality outcomes	Consistent with the high discriminatory performance of SOFA in the present study
Rudd et al. [7]	Adults with suspected infection in LMICs	qSOFA	qSOFA identified patients at increased risk of mortality	Supports the potential screening value of qSOFA observed in the present cohort
Serafim et al. [9]	Patients with suspected sepsis	qSOFA, SIRS	qSOFA demonstrated moderate diagnostic performance compared with other screening criteria	Consistent with the lower overall discrimination observed with qSOFA in the present study
Aarvold et al. [8]	Septic obstetric patients	MODS, Sepsis in Obstetrics Score	Organ dysfunction-based assessment demonstrated good prognostic performance	Supports the potential value of organ dysfunction-based assessment in obstetric patients
Present study	High-risk obstetric patients (n=188)	SOFA, qSOFA, omSOFA, omqSOFA	SOFA had the numerically highest AUC (0.97), followed by omSOFA (0.93); qSOFA and omqSOFA demonstrated high sensitivity and NPV at a cutoff ≥2	Suggests potential prognostic value of conventional and obstetrically modified organ dysfunction scores

Limitations

This study has several limitations. First, it was conducted at a single tertiary care center, which may limit the generalizability of the findings, particularly given the heterogeneous spectrum of obstetric and medical conditions represented in the study population. Second, only 15 maternal deaths occurred, limiting the precision of mortality-related diagnostic performance estimates, particularly sensitivity, specificity, PPV, and NPV. Third, the optimal cutoff values were derived and evaluated in the same cohort without internal or external validation, which may have resulted in optimistic estimates of diagnostic performance. These cutoff values should therefore be regarded as exploratory rather than definitive and require validation in independent populations. Fourth, formal pairwise statistical comparisons of the correlated ROC curves were not performed; therefore, differences in AUCs between the four scoring systems should be interpreted descriptively and not as evidence of statistical superiority. Finally, the single-center referral population and heterogeneity of the underlying clinical conditions may further limit applicability to other obstetric settings. Larger multicenter studies with adequate numbers of mortality events and independent validation cohorts are required to confirm these findings and establish a clinically applicable threshold.

## Conclusions

Among high-risk obstetric patients, SOFA demonstrated the numerically highest discriminatory performance for predicting maternal mortality, followed by obstetrically modified SOFA. Although qSOFA and omqSOFA showed lower specificity, their high sensitivity and negative predictive value in this cohort suggest potential value as bedside screening tools for early risk identification. However, given the small number of maternal deaths and the lack of internal or external validation, these findings and the derived cutoff values should be considered exploratory. Larger multicentre studies with independent validation are required to establish the clinical utility of these scoring systems in diverse obstetric populations.
